# MicroRNA Dysregulation and Hepatic Involvement in Alcohol-Related Macrocytic Anaemia: Links to Severity Without Predictive Value for Treatment Response

**DOI:** 10.3390/life16060918

**Published:** 2026-05-29

**Authors:** Corina Porr, Anca Vidrighin, Cristian Ichim, Samuel Bogdan Todor, Cosmina Diaconu, Paula Anderco

**Affiliations:** Faculty of Medicine, Lucian Blaga University of Sibiu, 550169 Sibiu, Romania; corina_sibiu@yahoo.com (C.P.); ancavidrighin@gmail.com (A.V.); cosmina_75dia@yahoo.com (C.D.); paula.anderco@ulbsibiu.ro (P.A.)

**Keywords:** alcohol-related macrocytic anemia, macrocytosis, chronic liver disease, microRNAs, treatment response

## Abstract

Background: Alcohol-related macrocytic anemia remains insufficiently characterized, particularly regarding molecular correlates of disease severity and treatment response. We evaluated the hepatic, inflammatory and microRNA profile of patients with alcohol-related macrocytic anemia and explored predictors of hematologic improvement. Methods: This prospective single-center observational study included 60 adults with alcohol-related macrocytic anemia. Patients were stratified by baseline severity (mild, n = 21; moderate, n = 17; severe, n = 22) and by treatment response (no change, n = 18; improved, n = 42). Hematologic, iron, nutritional, inflammatory, hepatic and miRNA (miR-21, miR-34a, miR-451a) data were analyzed using non-parametric tests, Spearman correlations and exploratory logistic regression. Results: Greater anemia severity was associated with longer alcohol exposure, higher weekly alcohol intake, higher GGT, more frequent chronic liver disease and higher expression of all three miRNAs. Hemoglobin and hematocrit decreased across severity groups, whereas MCV increased. Vitamin B12, folate, iron indices, CRP and IL-6 did not differ significantly by severity. No variable significantly distinguished patients who improved from those without hematologic change. All three miRNAs correlated inversely with hemoglobin and positively with MCV, GGT and alcohol-consumption measures. Conclusions: Alcohol-related macrocytic anemia shows a severity gradient linked to alcohol burden, hepatic involvement and distinct miRNA dysregulation. Predictors of treatment response were weak, supporting larger validation studies.

## 1. Introduction

Macrocytosis and macrocytic anemia are frequent hematologic findings in adults, and alcohol use is consistently recognized as one of the leading causes encountered in clinical practice [1,2]. In hospital-based series, alcohol and liver disease are among the most common etiologies of elevated mean corpuscular volume (MCV), while macrocytosis and macrocytic anemia have been reported in a substantial proportion of alcohol-dependent patients [3,4,5]. The mechanisms underlying alcohol-related macrocytic anemia are complex and extend beyond isolated vitamin deficiency [6]. Chronic ethanol exposure may impair hematopoiesis through direct bone marrow toxicity, altered erythroid precursor maturation, oxidative stress, membrane abnormalities and acetaldehyde-related injury [7]. In parallel, alcohol misuse may coexist with folate depletion, functional cobalamin disturbance, liver injury and other nutritional or metabolic abnormalities, resulting in a mixed hematologic phenotype [8,9,10,11]. Importantly, macrocytosis has also been documented in alcohol-exposed patients without folate deficiency, supporting a direct non-megaloblastic effect of alcohol itself [12]. From a diagnostic perspective, alcohol-related macrocytic anemia remains difficult to characterize because alcohol intake is often underreported, while indirect biomarkers such as MCV and gamma-glutamyl transferase (GGT) are informative but imperfect when interpreted alone [13,14]. This creates a need for integrated clinical-biological assessment and for additional markers capable of reflecting both alcohol-related tissue injury and disturbed erythropoiesis.

MicroRNAs (miRNAs) are small non-coding RNAs involved in inflammation, fibrosis, apoptosis and cellular differentiation, and they have emerged as potential biomarkers in liver disease and hematologic dysfunction [15,16,17]. miR-21 has been linked to alcohol-related liver injury and inflammatory-fibrogenic signaling, while miR-34a has been associated with alcohol-induced hepatic inflammation, angiogenesis and fibrosis severity [18,19]. By contrast, miR-451a is closely related to erythroid maturation and protection against oxidative stress [20,21]. However, the combined relevance of these miRNAs in alcohol-related macrocytic anemia remains insufficiently defined.

The novelty of the present study lies in the integrated assessment of hematologic, hepatic, inflammatory and miRNA profiles in patients with alcohol-related macrocytic anemia, stratified by both disease severity and treatment response. The study aimed to determine whether miR-21, miR-34a and miR-451a are associated with anemia severity and whether baseline clinical or molecular markers can predict short-term hematologic improvement.

## 2. Materials and Methods

### 2.1. Study Design, Setting and Ethics

This prospective, single-center observational study was conducted at the County Clinical Emergency Hospital Sibiu, Romania, over a 3-year period. The study aimed to characterize the clinical, hematological, biochemical, inflammatory and molecular features of alcohol-related macrocytic anemia and to examine baseline factors associated with anemia severity and treatment response. Adult patients consecutively evaluated in the hematology and internal medicine setting during the study period were screened for eligibility.

The study was conducted in accordance with the Declaration of Helsinki and local institutional requirements governing research involving human participants. Biological samples used for miRNA analysis were processed under anonymized conditions, and molecular analyses were performed only on coded samples.

### 2.2. Patient Selection and Eligibility Criteria

Patients aged 18 years or older were eligible for inclusion if they presented with anemia and a clinical-biological profile compatible with alcohol-related macrocytic anemia. Case identification was based on an integrated evaluation including direct patient interview, collateral history obtained from family members when available, clinical examination, routine laboratory findings and the overall medical record. To be included in the final cohort, patients were required to have complete baseline clinical and laboratory assessment, including hematological indices, iron status, vitamin B12 and folate levels, inflammatory markers, liver function tests and expression data for miR-21, miR-34a and miR-451a. In addition, follow-up data had to be available to permit classification of treatment response.

Patients were excluded if an alternative dominant cause of anemia or macrocytosis was evident, including acute or ongoing hemorrhage, known hematologic malignancy, current cytotoxic therapy, overt hemolytic anemia, advanced renal disease with a likely independent contribution to anemia, pregnancy, severe acute infection, recent transfusion capable of substantially altering baseline hematologic indices or incomplete baseline or follow-up data. Patients in whom macrocytosis was more plausibly attributable to a non-alcohol-related primary disorder were also excluded.

### 2.3. Definition of Alcohol-Related Macrocytic Anemia and Study Outcomes

Alcohol-related macrocytic anemia was defined by the coexistence of anemia, macrocytosis and clinically relevant alcohol exposure, after exclusion of other major causes of anemia or macrocytosis. Macrocytosis was defined as mean corpuscular volume > 100 fL. Clinically relevant alcohol exposure was defined as sustained alcohol intake for at least 6 months, documented by direct patient interview, collateral family history when available and review of the medical record. Supportive biochemical evidence included elevated GGT and/or compatible alcohol-related liver enzyme abnormalities, although these findings were considered supportive rather than mandatory diagnostic criteria. Competing causes of anemia were systematically excluded through clinical history, physical examination, complete blood count, reticulocyte count, iron studies, serum vitamin B12, serum folate, inflammatory markers, renal function assessment and review of active bleeding, hemolysis, hematologic malignancy, cytotoxic therapy, pregnancy, severe acute infection and recent transfusion.

Patients were classified according to baseline hemoglobin concentration into three predefined severity groups: mild anemia, hemoglobin ≥ 11.0 g/dL; moderate anemia, hemoglobin 9.0–10.9 g/dL; and severe anemia, hemoglobin < 9.0 g/dL. Based on this classification, the cohort included 21 patients with mild anemia, 17 with moderate anemia and 22 with severe anemia.

After baseline assessment, all patients received standard-of-care management individualized according to clinical status and the treating physician’s judgment. Therapeutic measures included alcohol cessation counseling, supportive care and correction of associated nutritional or metabolic abnormalities when clinically indicated. Patients were reassessed during follow-up and treatment response was categorized as improved or no change. Improvement was defined quantitatively as an increase in hemoglobin of at least 1.0 g/dL from baseline and/or an increase in hematocrit of at least 3 percentage points, together with stabilization or reduction of MCV. Patients who did not meet these criteria were classified as having no change. This definition was applied before statistical comparison between response groups.

### 2.4. Data Collection and Baseline Assessments

The following baseline variables were collected for all participants: age, sex, ethnicity, body mass index (BMI), Charlson Comorbidity Index, chronic liver disease status, serum albumin and weight loss during the preceding 6 months. Chronic liver disease was recorded on the basis of documented prior diagnosis and/or compatible clinical, imaging and biochemical findings noted in the medical record. Alcohol exposure was further quantified using two continuous variables: years of alcohol consumption and average weekly alcohol consumption, expressed in grams of ethanol per week. Duration of alcohol use reflected sustained exposure over time, while weekly intake captured current or recent consumption burden. Information was obtained primarily from direct anamnesis and cross-checked against collateral family history when available.

Baseline venous blood samples were collected at enrollment as part of the routine diagnostic work-up. All hematological and biochemical measurements were performed according to standardized operating procedures and internal quality-control requirements. The hematological panel included hemoglobin, hematocrit, mean corpuscular volume, red cell distribution width, reticulocyte count, white blood cell count and platelet count. Iron metabolism was evaluated using serum iron, ferritin, total iron-binding capacity and transferrin saturation. Nutritional and inflammatory status was assessed by serum vitamin B12, serum folate, C-reactive protein, interleukin-6, serum albumin and documented recent weight loss. Liver-related biochemical assessment included gamma-glutamyl transferase, alanine aminotransferase and aspartate aminotransferase. All laboratory values were interpreted according to the reference ranges in use at the study institution during the study period.

Baseline expression profiling of miR-21, miR-34a and miR-451a was performed on whole-blood-derived samples collected at enrollment, before treatment-response categorization. Total RNA enriched for small non-coding RNA species was isolated using a validated small-RNA extraction protocol. Reverse transcription was performed using a miRNA-specific reverse transcription protocol, followed by quantitative real-time polymerase chain reaction for miR-21, miR-34a and miR-451a. Relative miRNA expression was normalized to U6 snRNA as the endogenous reference control and calculated using the comparative Ct method. All qPCR reactions were performed in triplicate, and the mean Ct value was used for analysis. Molecular analyses were performed blinded to the final treatment-response category.

Only patients with complete baseline and follow-up data for the variables included in the principal analyses were retained in the final dataset. Accordingly, the study was analyzed using a complete-case approach. The final analytical cohort consisted of 60 patients. For the severity-based analysis, participants were stratified into mild (n = 21), moderate (n = 17) and severe (n = 22) anemia groups. For the treatment-response analysis, patients were classified as showing no change (n = 18) or improvement (n = 42).

### 2.5. Statistical Analysis

Statistical analysis was performed using standard medical statistical software (SPSS software, version 24.0). Continuous variables were summarized as median and interquartile range (IQR), while categorical variables were presented as absolute frequencies and percentages. Because most continuous variables showed non-normal distribution, non-parametric methods were used throughout. For comparisons across the three anemia-severity groups, the Kruskal-Wallis test was applied to continuous variables and the chi-square test or Fisher’s exact test to categorical variables, as appropriate. For comparisons between treatment-response groups, continuous variables were analyzed using the Mann-Whitney U test and categorical variables using the chi-square test or Fisher’s exact test, as appropriate. Correlations between serum vitamin B12 or folate levels and miRNA expression were assessed using Spearman’s rank correlation coefficient. Spearman analysis was also used to evaluate associations between each miRNA marker and the available continuous clinical and laboratory variables, including hematological indices, iron studies, inflammatory markers, liver enzymes and alcohol-consumption parameters. Scatter plots were generated with bootstrapped 95% confidence interval bands. To reduce the risk of false-positive findings due to multiple correlation analyses, false-discovery-rate adjustment using the Benjamini–Hochberg method was applied to the exploratory miRNA correlation analyses. Both unadjusted and adjusted significance levels were considered, and correlations were interpreted cautiously when they did not remain significant after correction. Binary logistic regression analysis was performed to examine baseline predictors of treatment response, with improvement versus no change as the dependent outcome. Candidate predictors were selected on the basis of biological relevance and signal observed in univariable analyses. Odds ratios and 95% confidence intervals were reported. Model performance was evaluated using the Akaike information criterion, Bayesian information criterion, McFadden pseudo-R^2^ and the Hosmer-Lemeshow goodness-of-fit test. To further assess potential clinical usefulness, decision curve analysis was performed across a range of threshold probabilities in order to compare the net benefit of progressively enriched predictive models. A two-sided *p* value < 0.05 was considered statistically significant. Given the exploratory nature of the correlation analyses, these findings were interpreted as hypothesis-generating. Because of the limited sample size and the number of exploratory comparisons, the molecular correlation analyses and treatment-response models were interpreted as hypothesis-generating rather than confirmatory. The possibility of false-positive findings due to multiple testing was considered when interpreting the results.

## 3. Results

### 3.1. Baseline Severity-Profile Assessment

The cohort comprised 60 patients stratified into three anemia-severity groups: mild (n = 21), moderate (n = 17) and severe (n = 22). The three groups were broadly comparable in terms of age, BMI and general nutritional indicators, with no statistically significant differences in serum albumin, recent weight loss or comorbidity burden as assessed by the Charlson Comorbidity Index (all *p* > 0.05). These findings suggest that the groups did not materially differ with respect to baseline nutritional status or overall comorbidity burden. By contrast, alcohol-exposure variables and liver-related parameters showed clearer between-group differences. Patients in the moderate and severe groups had longer duration of alcohol consumption and higher average weekly alcohol intake than those in the mild group (both *p* < 0.001), supporting a gradient of alcohol-related disease burden across anemia-severity categories.

Hematological parameters demonstrated a clear gradient across the three-baseline severity-profile groups. Hemoglobin declined progressively from the mild to the severe group (*p* < 0.001), mirrored by corresponding decreases in hematocrit. Mean corpuscular volume (MCV) was markedly elevated in the moderate and severe groups compared with the mild group (*p* < 0.001), indicating that macrocytosis clustered predominantly in patients with more advanced hematologic impairment.

Iron studies were uniformly non-significant across groups. Serum iron, ferritin and TIBC did not differ significantly between the mild, moderate and severe baseline severity-profile groups (all *p* > 0.05), arguing against a major contribution of iron deficiency within this cohort. Similarly, serum B12 and folate levels, as well as inflammatory markers CRP and IL-6, did not differ significantly, suggesting that nutritional deficiency of these micronutrients and systemic inflammation do not independently stratify by severity in this population.

Among liver function tests, GGT was markedly elevated in the moderate (121.90 U/L) and severe (121.10 U/L) groups relative to mild anemia (39.10 U/L, *p* < 0.001), consistent with chronic alcohol-related hepatic injury. ALT and AST did not reach statistical significance, suggesting subclinical or predominantly cholestatic hepatic involvement rather than overt hepatocellular damage in this sample.

All three miRNA markers: miR-21, miR-34a and miR-451a, showed significantly higher expression in the moderate and severe groups compared to mild anemia (all *p* < 0.001). Expression levels of miR-34a showed a progressive increase from mild (1.05) through moderate (1.78) to severe (2.11), while miR-21 and miR-451a showed a step-up from mild to moderate with a plateau into severe disease.

Regarding categorical variables, sex distribution was comparable across groups (*p* = 0.365). A significant difference in ethnicity was observed (*p* = 0.010), with the mild group containing a higher proportion of non-Caucasian patients (28.6%) compared to near-absent proportions in the moderate and severe groups. Chronic liver disease was significantly more prevalent with increasing severity (0% in mild, 17.6% in moderate, 40.9% in severe; *p* = 0.003), corroborating the hepatic injury findings from GGT.

### 3.2. Treatment Response Assessment

Table 1 presents the characteristics of 60 patients stratified by treatment response: those showing no change (n = 18) and those who improved (n = 42). Unlike the severity-based analysis in Table 1, no variable reached statistical significance in distinguishing treatment responders from non-responders, a finding that is clinically informative and warrants detailed discussion. According to these predefined criteria, 42 patients were classified as improved and 18 as no change.

Demographic and anthropometric variables including age, BMI and comorbidity index were comparable between groups. While the no-change group was numerically older (median 52.5 vs. 45.5 years), this difference did not reach significance (*p* = 0.160), possibly reflecting limited statistical power given the sample size. Alcohol consumption parameters, years of use and weekly quantity, were also similar between groups, indicating that the burden of alcohol exposure per se does not determine treatment outcome once patients have been enrolled for management.

Hematological parameters at baseline were strikingly similar between responders and non-responders. Hemoglobin, hematocrit, MCV, RDW, reticulocyte count, WBC and platelets showed no significant between-group differences (all *p* > 0.05). This uniformity suggests that baseline anemia severity did not independently predict the likelihood of treatment response, a finding supported by the subgroup distribution shown at the bottom of Table 2, where mild, moderate and severe anemia were similarly represented in the two response groups.

Iron studies, nutritional markers and inflammatory parameters were all non-significant between groups. The absence of differences in serum vitamin B12 (*p* = 0.345) and folate (*p* = 0.567) between responders and non-responders is consistent with the findings in Table 1, suggesting that these micronutrients did not materially influence anemia severity or treatment response in this cohort. Among liver function tests, GGT was numerically higher in the no-change group than in the improved group, but this difference did not reach statistical significance (111 U/L vs. 83.70 U/L, *p* = 0.140). Therefore, baseline GGT did not significantly distinguish treatment-response categories in this cohort. This pattern is biologically plausible, as persistent hepatic dysfunction may impair erythropoietic recovery and it may warrant further evaluation in larger cohorts. ALT and AST did not differ between groups.

The expression levels of miR-21, miR-34a and miR-451a did not differ significantly between response groups. Although miR-451a was numerically higher in the no-change group, this difference was not statistically significant. Therefore, the present data do not support the use of baseline miRNA expression as a predictor of short-term hematologic response.

Categorical variables including sex, ethnicity, anemia severity and chronic liver disease did not significantly distinguish responders from non-responders. The absence of significant predictors in this analysis underscores the multifactorial and individually variable nature of treatment response in alcohol-related macrocytic anemia and highlights the need for multivariable predictive modeling rather than reliance on any single clinical or laboratory feature.

**Figure 1:**
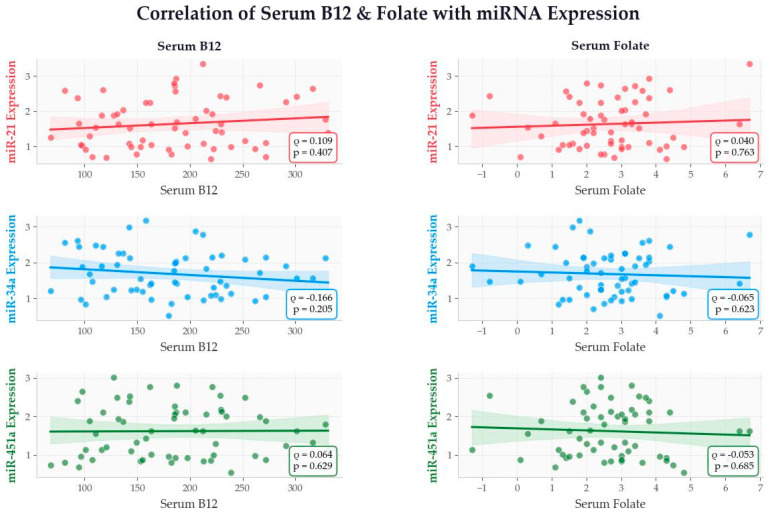
Correlation of serum B12 and folate with miRNA expression. Fitted lines include bootstrapped 95% confidence intervals. Correlation coefficients are Spearman rho values.

Spearman correlation analyses were performed between serum B12 and serum folate levels and the expression of miR-21, miR-34a and miR-451a. Scatter plots with bootstrapped 95% confidence interval bands are provided in Figure 1.

Neither serum B12 nor serum folate showed a statistically significant correlation with any of the three miRNA markers. For miR-21, the correlations with B12 (rho = 0.109, *p* = 0.407) and folate (rho = 0.040, *p* = 0.763) were negligible and non-significant. For miR-34a, correlations were weakly negative but again non-significant (B12: rho = −0.166, *p* = 0.205; Folate: rho = −0.065, *p* = 0.623). miR-451a similarly showed no meaningful association with either vitamin (B12: rho = 0.064, *p* = 0.629; Folate: rho = −0.053, *p* = 0.685).

**Figure 2:**
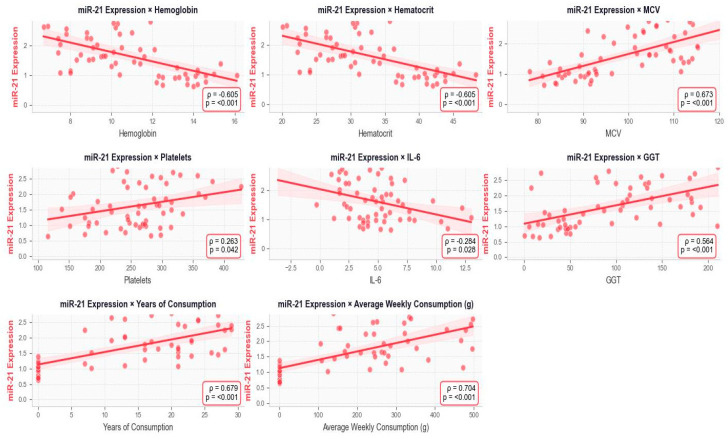
Spearman correlations of miR-21 expression with significant clinical and laboratory variables. Fitted lines include bootstrapped 95% confidence intervals.

**Figure 3:**
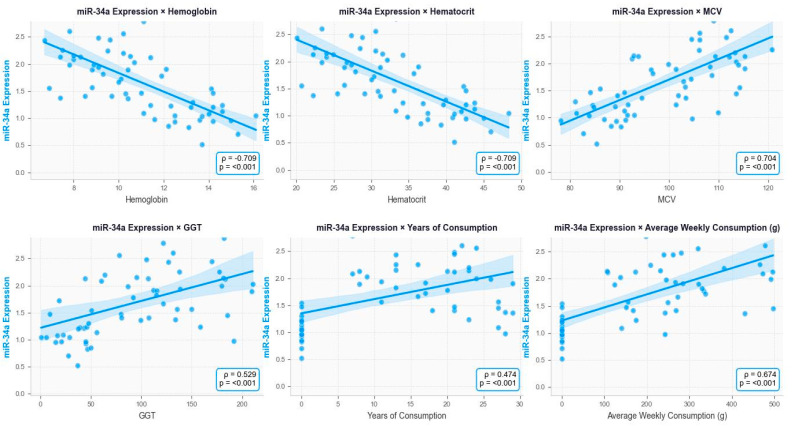
Spearman correlations of miR-34a expression with significant clinical and laboratory variables. Fitted lines include bootstrapped 95% confidence intervals.

**Figure 4:**
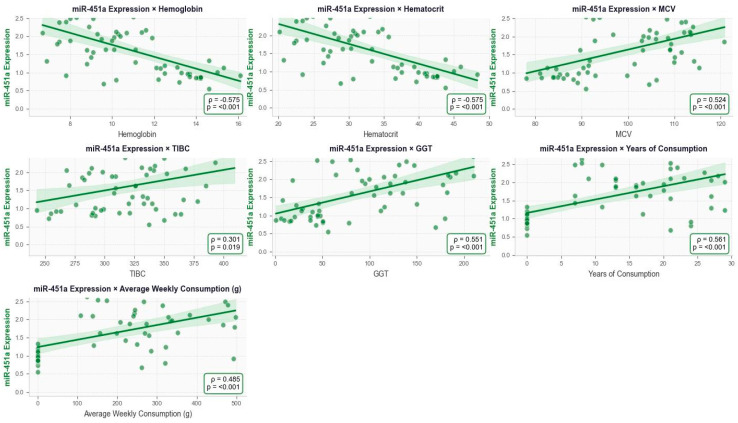
Spearman correlations of miR-451a expression with significant clinical and laboratory variables. Fitted lines include bootstrapped 95% confidence intervals.

Spearman correlation analyses were performed between each miRNA and all available continuous clinical variables, including hematological parameters, iron studies, liver function tests, inflammatory markers and alcohol consumption indices. Correlation analyses were interpreted after false-discovery-rate adjustment using the Benjamini–Hochberg method. Associations that remained significant after adjustment were considered more robust, whereas nominally significant unadjusted associations were interpreted as exploratory. Scatter plots for all significant correlations are provided in Figure 2, Figure 3 and Figure 4.

miR-21 expression demonstrated significant correlations with eight clinical variables (Figure 2). The strongest associations were observed with markers of alcohol consumption: average weekly consumption (rho = 0.704, *p* < 0.001) and years of consumption (rho = 0.679, *p* < 0.001), indicating that cumulative alcohol exposure is closely linked to miR-21 upregulation. Among hematological parameters, miR-21 was strongly inversely correlated with both hemoglobin (rho = −0.605, *p* < 0.001) and hematocrit (rho = −0.605, *p* < 0.001), reflecting its consistent elevation in the context of worsening anemia. A significant positive correlation with MCV (rho = 0.673, *p* < 0.001) confirms the association between miR-21 upregulation and alcohol-related macrocytic anemia. GGT showed a strong positive correlation with miR-21 (rho = 0.564, *p* < 0.001), consistent with a shared dependence on alcohol-induced hepatic injury. A weaker but significant positive association was found with platelet count (rho = 0.263, *p* = 0.042) and a modest inverse correlation was observed with IL-6 (rho = −0.284, *p* = 0.028), a finding which may reflect compensatory anti-inflammatory signalling mediated by miR-21 in the context of chronic alcohol exposure.

miR-34a showed the strongest overall correlations among the three miRNA markers (Figure 3). Inverse associations with hemoglobin (rho = −0.709, *p* < 0.001) and hematocrit (rho = −0.709, *p* < 0.001) were the largest in magnitude across the entire correlation analysis, suggesting that miR-34a expression is most tightly coupled to the degree of erythrocyte depletion. A strong positive correlation with MCV (rho = 0.704, *p* < 0.001) further supports its role as a marker of macrocytic erythroid disruption. miR-34a was also significantly correlated with GGT (rho = 0.529, *p* < 0.001), reinforcing the hepatic injury axis. Alcohol exposure variables were again significant predictors: average weekly consumption (rho = 0.674, *p* < 0.001) and years of consumption (rho = 0.474, *p* < 0.001). Notably, miR-34a did not correlate significantly with platelet count or IL-6, distinguishing its correlation profile from miR-21 and suggesting that its upregulation is more specifically tied to erythropoietic and hepatic pathways rather than broader inflammatory or thrombocytic mechanisms.

miR-451a exhibited the broadest correlation profile of the three markers, with seven significant associations (Figure 4). As with the other miRNAs, inverse correlations with hemoglobin (rho = −0.575, *p* < 0.001) and hematocrit (rho = −0.575, *p* < 0.001) and a positive correlation with MCV (rho = 0.524, *p* < 0.001) were highly significant, confirming a consistent pattern of erythroid disruption across all three markers. A unique finding for miR-451a was its significant positive correlation with TIBC (rho = 0.301, *p* = 0.019), which was not significant for miR-21 or miR-34a. TIBC is a marker of iron transport capacity and its elevation is associated with iron-deficient erythropoiesis; this association may reflect a distinct regulatory role of miR-451a in erythroid iron metabolism, consistent with its known function in red cell maturation. GGT (rho = 0.551, *p* < 0.001), years of consumption (rho = 0.561, *p* < 0.001) and average weekly consumption (rho = 0.485, *p* < 0.001) were all significant, maintaining the alcohol-miRNA relationship seen across all three markers.

### 3.3. Logistic Regression Analysis

The binary logistic regression model evaluated four candidate predictors of treatment response: GGT, miR-451a expression, age and miR-34a expression, with improvement versus no change as the outcome. The overall model demonstrated acceptable calibration, as evidenced by a non-significant Hosmer-Lemeshow statistic (χ^2^ = 4.162, *p* = 0.842), indicating that predicted probabilities aligned well with observed outcomes across risk groups. However, model discrimination was modest, reflected by a McFadden pseudo-R^2^ of 0.064, which suggests that the included predictors collectively explained only a limited proportion of the variance in treatment response.

None of the four predictors reached statistical significance at the conventional threshold (*p* < 0.05). GGT showed an odds ratio near unity (OR = 0.995, 95% CI: 0.984–1.007, *p* = 0.450), indicating no meaningful association between hepatic injury severity and the likelihood of treatment response. miR-451a expression (OR = 0.777, *p* = 0.631) and miR-34a expression (OR = 0.764, *p* = 0.625) both showed odds ratios below 1.0, suggesting a directional trend toward lower response probability with higher expression, but confidence intervals were wide and crossed 1.0, precluding any definitive conclusion. Age similarly showed no significant predictive value (OR = 0.967, *p* = 0.237).

The AIC (78.622) and BIC (89.093) provide a benchmark for comparison with alternative or more parsimonious models in future analyses.

**Figure 5:**
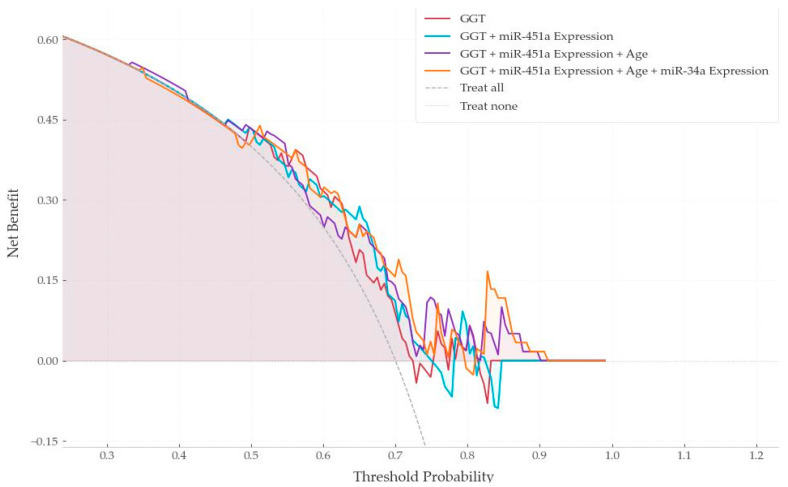
Exploratory decision curve analysis. Given the limited model performance and absence of significant predictors, this analysis should be interpreted as hypothesis-generating only.

Decision curve analysis was retained only as an exploratory assessment of model behavior. Because no predictor was statistically significant and model performance was limited, the DCA results should not be interpreted as evidence of clinical usefulness or as support for implementation in treatment decision-making (Figure 5).

In the clinically relevant range of threshold probabilities (approximately 0.25 to 0.50), all four models demonstrate a positive net benefit that exceeds both the “treat all” (dashed grey) and “treat none” (dotted grey) reference strategies. This is an encouraging finding, indicating that each model, even GGT alone, would offer some net clinical value if used to guide treatment decisions at moderate to moderately-high probability thresholds. The convergence of all four curves in this lower range reflects the predominantly similar predictive signal captured by GGT across models, consistent with the logistic regression findings in Table 3 where GGT contributed most substantially to model structure, albeit without individual significance.

Notably, the models incorporating miRNA expression variables, particularly GGT + miR-451a Expression + Age (purple curve) and the full model with miR-34a Expression (orange curve), demonstrate a marginally superior net benefit at threshold probabilities between 0.45 and 0.65, maintaining positive net benefit at higher thresholds where GGT alone begins to lose advantage over the “treat all” strategy. This pattern suggests that the addition of miRNA markers and age modestly extends the clinically useful range of the predictive model, even if individual coefficients did not reach statistical significance in the logistic regression. This is consistent with known limitations of *p*-value thresholds in small samples and supports the hypothesis that a multi-marker approach incorporating molecular biomarkers holds additive clinical value.

Above a threshold probability of approximately 0.65–0.70, the net benefit of all models becomes erratic and crosses below zero, most prominently for the GGT + miR-451a model (cyan curve) which dips substantially into negative territory. This instability at high thresholds is expected in DCA when sample sizes are small, as there are few observations at the extreme tail of predicted probabilities, resulting in volatile net benefit estimates. It should not be interpreted as evidence of harm from model use, but rather as an artefact of limited statistical power in the upper probability range. Clinical application of these models should therefore be restricted to the moderate threshold range (0.30–0.60) where curves are stable and positive. Overall, Table 4 shows that disease severity was mainly associated with alcohol burden, hepatic involvement and miRNA expression, whereas treatment response could not be predicted from baseline hematologic, biochemical, hepatic or molecular variables.

## 4. Discussion

This prospective single-center study suggests that alcohol-related macrocytic anemia is associated with alcohol burden, hepatic involvement and altered miRNA expression. More severe anemia was linked to longer alcohol exposure, higher weekly alcohol intake, higher GGT values and more frequent chronic liver disease. In parallel, miR-21, miR-34a and miR-451a expression increased across severity groups. In contrast, iron indices, vitamin B12, folate, CRP and IL-6 did not significantly differ across severity groups, and no baseline variable significantly predicted short-term hematologic improvement.

The graded association between alcohol exposure and anemia severity supports the role of chronic ethanol toxicity in this condition. Patients with moderate and severe anemia had longer alcohol exposure and higher weekly ethanol intake than those with mild anemia. The parallel increase in GGT and chronic liver disease prevalence further supports a hepato-hematologic pattern of injury [3,6,22].

Hemoglobin and hematocrit declined progressively, whereas MCV was significantly higher in the moderate and severe groups than in the mild group, confirming that macrocytosis clusters with more advanced hematologic impairment. At the same time, the absence of major between-group differences in RDW, reticulocyte count, leukocyte count, platelet count and iron indices suggests that the dominant process in this cohort was not an overt regenerative anemia, major iron-restricted erythropoiesis or diffuse marrow failure. Prior studies support this interpretation by showing that chronic alcohol exposure can induce macrocytosis through direct toxic effects on erythroid precursors and through acetaldehyde-related marrow injury, including vacuolization of pronormoblasts and ring sideroblast formation [23,24,25].

An important negative finding was the lack of significant stratification by serum vitamin B12, folate, CRP or IL-6. These data do not exclude contributory micronutrient insufficiency at the individual level, but they do indicate that baseline severity in this cohort was not primarily driven by measurable differences in these parameters. This is an important point, because alcohol-related macrocytosis is often reflexively attributed to folate deficiency alone. In contrast, previous clinical studies have demonstrated macrocytosis in alcohol-exposed patients even in the absence of folate deficiency and current laboratory reviews emphasize that ethanol and acetaldehyde exert direct toxic effects on hematopoietic precursor cells and red-cell morphology independent of classic megaloblastic deficiency states [12,23,26,27]. Our findings therefore favor a predominantly toxic and hepato-metabolic model of severity stratification in this population.

The liver-related findings merit separate comment: GGT being the only liver enzyme that differed significantly across the severity groups, while AST and ALT did not. This pattern may reflect the fact that GGT is particularly responsive to sustained alcohol exposure and microsomal enzyme induction, whereas transaminases are less reliable as isolated markers of chronic drinking burden and may remain relatively modest outside overt hepatocellular injury. The absence of significant AST and ALT differences should therefore not be interpreted as evidence against hepatic involvement. Rather, in the present cohort, GGT appears to have captured the chronic alcohol-related hepatic signal more effectively than transaminases, which is coherent with broader literature on indirect alcohol biomarkers [28,29,30].

A major contribution of this study is the demonstration that all three analyzed miRNAs were significantly upregulated in patients with more severe disease. Among them, miR-34a showed the most coherent gradient, increasing from mild to moderate to severe anemia and displaying the strongest inverse correlations with hemoglobin and hematocrit. This pattern is biologically plausible. Experimental work in alcohol-associated liver disease has shown elevated miR-34a expression together with links to macrophage-associated inflammation, angiogenesis and fibrosis [19,31,32]. In the context of our data, the strong association of miR-34a with both worsening anemia and higher GGT suggests that this marker may capture the convergence of hepatic injury and erythroid disruption particularly well.

miR-21 and miR-451a showed a somewhat different behavior, with a marked increase from mild to moderate disease followed by a relative plateau in severe disease. For miR-21, this may indicate that its induction occurs relatively early once a threshold of alcohol-related tissue injury is reached, after which additional clinical deterioration is not mirrored by a strictly linear rise. This interpretation is compatible with prior evidence that miR-21 is dysregulated in alcohol-related liver injury and participates in apoptosis regulation, inflammatory signaling and fibrogenic pathways [33,34,35]. For miR-451a, the observed pattern is especially interesting because this miRNA is tightly linked to erythroid maturation and protection from oxidant stress. Its elevation in patients with more severe macrocytic anemia may therefore reflect compensatory or stress-related erythroid signaling in the setting of alcohol-induced ineffective erythropoiesis.

All three miRNAs correlated inversely with hemoglobin and hematocrit and positively with MCV, GGT and alcohol-exposure variables, indicating that they are not random epiphenomena but are closely aligned with the central clinical axes of this disease. The particularly strong association of miR-21 and miR-34a with alcohol intake variables suggests that these markers may be responsive to cumulative alcohol-related toxicity, whereas the association of miR-451a with TIBC raises the possibility of a more specific relationship with erythroid iron handling or stress erythropoiesis. By contrast, the absence of significant correlations between these miRNAs and serum B12 or folate supports the notion that the miRNA signature identified here is not simply a surrogate for vitamin deficiency, but may reflect a distinct alcohol-hepatic-erythroid regulatory network.

From a functional perspective, the three miRNAs analyzed in this study are linked to pathways relevant to alcohol-related hepatic and erythroid injury. miR-21 has been associated with inflammatory and fibrogenic signaling, including PTEN/Akt and NF-κB-related pathways. miR-34a is linked to hepatocyte injury, apoptosis, cellular senescence and macrophage-associated inflammatory responses in alcohol-related liver disease. miR-451a is closely related to erythroid maturation and protection against oxidative stress during erythropoiesis. These known biological roles support the plausibility of the observed associations with GGT, hemoglobin, hematocrit and MCV. However, no functional validation was performed in the present study; therefore, these interpretations remain exploratory.

In contrast to the severity analysis, baseline characteristics did not significantly distinguish patients who improved from those who showed no change. Short-term hematologic recovery may depend on dynamic post-baseline factors not fully captured here, including adherence to alcohol cessation, nutritional rehabilitation, reversibility of marrow suppression, hepatic recovery and timing of reassessment. Therefore, the present results do not support the clinical use of miRNA markers or liver enzymes as predictors of treatment response [28,36,37].

The regression analysis is consistent with this interpretation. Although the model showed acceptable calibration, none of the included variables independently predicted improvement and the explained variance was low. Still, the decision curve analysis suggests that models incorporating GGT and miRNA markers may retain modest clinical usefulness within intermediate threshold ranges, even in the absence of individually significant coefficients. For now, these findings should be interpreted cautiously and as supportive of further model development rather than as evidence for immediate clinical implementation.

This study has several strengths. It prospectively evaluated a clearly defined cohort, integrated hematologic, biochemical, inflammatory, hepatic and molecular data, and approached alcohol-related macrocytic anemia as a multidimensional disorder rather than as an isolated blood count abnormality. At the same time, several limitations must be acknowledged. The study was single-center and involved a relatively small sample, which limits statistical power, especially for treatment-response modeling. The relatively large number of exploratory comparisons further increases the risk of false-positive findings, particularly for miRNA correlations; therefore, these molecular associations should be interpreted as hypothesis-generating and require validation in independent cohorts. Alcohol exposure was assessed clinically rather than by direct biomarkers such as phosphatidylethanol or ethyl glucuronide. Response was dichotomized into no change versus improvement, which is clinically practical but may obscure more nuanced trajectories of hematologic recovery. The relatively small sample size and the number of exploratory comparisons increase the risk of type I error, particularly for miRNA correlations. Therefore, the molecular associations reported in this study should be interpreted as hypothesis-generating and require validation in larger independent cohorts. The absence of a healthy control group and of an alcohol-exposed group without anemia is an important limitation. Therefore, the observed miRNA upregulation cannot be attributed exclusively to anemia severity, alcohol toxicity or liver disease. The findings should instead be interpreted as associations within a cohort of patients with alcohol-related macrocytic anemia. Correlation analyses were interpreted after false-discovery-rate adjustment using the Benjamini–Hochberg method. Associations that remained significant after adjustment were considered more robust, whereas nominally significant unadjusted associations were interpreted as exploratory. Finally, because follow-up was observational and management was individualized, residual heterogeneity in treatment intensity, abstinence behavior and timing of reassessment is unavoidable.

## 5. Conclusions

This study supports three main conclusions. First, alcohol-related macrocytic anemia shows a severity gradient associated with alcohol burden, hepatic involvement and altered miRNA expression. Second, miR-21, miR-34a and miR-451a were associated with anemia severity and correlated with hemoglobin, hematocrit, MCV, GGT and alcohol exposure, suggesting potential value as markers of disease phenotype. Third, baseline miRNA expression and liver-related markers did not significantly predict short-term hematologic response. From a clinical perspective, these findings suggest that miRNA profiling may help characterize disease severity but cannot currently be used to guide treatment-response prediction. Future studies should include larger multicenter cohorts, healthy controls, alcohol-exposed patients without anemia, objective alcohol biomarkers, standardized treatment protocols, predefined response criteria and serial miRNA measurements before and after treatment.

## Figures and Tables

**Table 1:** General Characteristics by Baseline Hematologic Severity Profile.

Variable	Mild (n = 21)	Moderate (n = 17)	Severe (n = 22)	*p*-Value
**Continuous variables—Median [IQR]**				
**Age (years)**	49 [42–57]	48 [43–54]	45.50 [35.25–55.25]	0.492
**BMI (kg/m^2^)**	23.7 [21.3–26]	24.9 [23.3–26.3]	25.8 [23.8–27.2]	0.457
**Charlson Comorbidity Index**	1 [1–2]	2 [1–3]	1 [1–2]	0.173
**Years of Alcohol Consumption**	8 [5–12]	21 [16–24]	19 [13–21.75]	<0.001
**Average Weekly Consumption (g)**	126 [84–168]	243 [141–338]	264.50 [234–327.25]	<0.001
**Serum Albumin (g/dL)**	3.59 [3.33–3.81]	3.70 [3.24–3.92]	3.53 [3.26–3.93]	0.867
**Weight Loss Past 6 Months (kg)**	1.60 [−0.20–2.70]	1.50 [−1.30–2.50]	1.55 [−0.38–3.60]	0.640
**Hematological parameters**				
**Hemoglobin (g/dL)**	12.4 [11.8–12.8]	10.5 [10.2–11.1]	8.4 [7.5–9.0]	<0.001
**Hematocrit (%)**	36.8 [35.1–38.4]	31.5 [30.6–33.3]	25.2 [22.7–27.2]	<0.001
**MCV (fL)**	101.4 [98.8–104.2]	108.6 [103–112.1]	105.25 [97.4–111.1]	<0.001
**RDW (%)**	15.7 [13.3–16.4]	14.2 [13.6–16.2]	14.7 [13–16.3]	0.849
**Reticulocyte Count (×10^9^/L)**	48 [37–68]	51 [40–58]	51.5 [42.2–57.7]	0.777
**WBC (×10^9^/L)**	5.7 [5.3–6.4]	6 [5.3–6.8]	6.05 [5.5–6]	0.745
**Platelets (×10^9^/L)**	264 [209–282]	257 [228–284]	282.50 [199.50–315]	0.423
**Iron (µg/dL)**	49.1 [38–53.2]	43.70 [34.7–54]	44.3 [37–52.3]	0.820
**Ferritin (ng/mL)**	22.6 [11.9–33.3]	21.2 [9.6–35.9]	28.4 [13.3–39]	0.631
**TIBC (µg/dL)**	312 [289–337]	306 [291–336]	336.5 [311–362]	0.079
**Nutritional and inflammatory markers**				
**Serum B12 (pg/mL)**	163 [120.6–219]	205 [185.1–228.6]	162.5 [133–242]	0.657
**Serum Folate (ng/mL)**	2.80 [1.80–4.10]	2.40 [2–3.30]	3.05 [1.90–3.48]	0.700
**CRP (mg/L)**	6.80 [4.80–12.60]	10.90 [2.70–15]	8.95 [4.38–12.62]	0.890
**IL-6 (pg/mL)**	4.80 [3.70–6.10]	5.30 [3.20–6.10]	3.65 [2.12–5.55]	0.195
**Liver function tests**				
**GGT (U/L)**	39 [20–46]	121 [79–182]	121 [97–138]	<0.001
**ALT (U/L)**	44 [26–57]	36 [30–57]	43 [35–48]	0.853
**AST (U/L)**	38 [25–46]	31 [25–35]	34 [26–45]	0.346
**miRNA expression (relative units)**				
**miR-21 Expression**	0.98 [0.78–1.05]	1.92 [1.45–2.72]	1.84 [1.55–2.25]	<0.001
**miR-34a Expression**	1.05 [0.95–1.23]	1.78 [1.45–2.14]	2.11 [1.84–2.40]	<0.001
**miR-451a Expression**	0.96 [0.86–1.11]	2.06 [1.88–2.18]	1.91 [1.58–2.41]	<0.001
**Sex**				
**Female**	8 (38.1%)	4 (23.5%)	10 (45.5%)	0.365
**Male**	13 (61.9%)	13 (76.5%)	12 (54.5%)	0.365
**Ethnicity**				
**Caucasian**	15 (71.4%)	17 (100.0%)	21 (95.5%)	0.010
**Other**	6 (28.6%)	0 (0.0%)	1 (4.5%)	0.010
**Chronic Liver Disease**				
**No**	21 (100.0%)	14 (82.4%)	13 (59.1%)	0.003
**Yes**	0 (0.0%)	3 (17.6%)	9 (40.9%)	0.003

Abbreviations: BMI, body mass index; MCV, mean corpuscular volume; RDW, red cell distribution width; WBC, white blood cell count; TIBC, total iron-binding capacity; CRP, C-reactive protein; IL-6, interleukin-6; GGT, gamma-glutamyl transferase; ALT, alanine aminotransferase; AST, aspartate aminotransferase; IQR, interquartile range. *p*-values generated by Kruskal-Wallis test (continuous variables) and Chi-square or Fisher’s exact test (categorical variables), as appropriate.

**Table 2:** General Characteristics by Treatment Response.

Variable	No Change (n = 18)	Improved (n = 42)	*p*-Value
**Continuous variables—Median [IQR]**			
**Age (years)**	52.50 [46–57.50]	45.50 [41.25–54]	0.160
**BMI (kg/m^2^)**	25.35 [22.38–27.82]	25.05 [22.85–26.27]	0.561
**Charlson Comorbidity Index**	1 [1–2]	1 [1–2]	0.642
**Years of Alcohol Consumption**	13 [1.75–22.50]	14.50 [0–21]	0.869
**Average Weekly Consumption (g)**	204 [26.50–368.75]	186.50 [0–268.50]	0.340
**Serum Albumin (g/dL)**	3.76 [3.05–3.96]	3.57 [3.28–3.86]	0.669
**Weight Loss Past 6 Months (kg)**	1.75 [0.90–2.50]	1.50 [−0.73–2.77]	0.617
**Hematological parameters**			
**Hemoglobin (g/dL)**	10.25 [8.12–12.05]	11.10 [9.10–13.28]	0.283
**Hematocrit (%)**	30.75 [24.38–36.15]	33.30 [27.30–39.83]	0.283
**MCV (fL)**	100.80 [91.9–112.8]	101.35 [89.1–108.9]	0.266
**RDW (%)**	14.95 [13.15–16.40]	15.05 [13.38–16.18]	0.968
**Reticulocyte Count (×10^9^/L)**	46.50 [37–60.75]	52.50 [42–63]	0.366
**WBC (×10^9^/L)**	5.65 [5.15–6]	6.20 [5.43–6.80]	0.119
**Platelets (×10^9^/L)**	278 [242–297.75]	262.50 [215.25–297.25]	0.321
**Iron studies**			
**Iron (µg/dL)**	47.40 [40.95–60.40]	45.55 [34.60–51.77]	0.313
**Ferritin (ng/mL)**	27.40 [8.20–38.67]	22.60 [11.75–33.58]	0.723
**TIBC (µg/dL)**	330 [297–347.75]	320 [290.50–341.50]	0.483
**Transferrin Saturation (%)**	13.20 [12.03–19.95]	13.75 [10.72–17.12]	0.354
**Nutritional and inflammatory markers**			
**Serum B12 (pg/mL)**	170.50 [124.57–208.38]	185.20 [135–228.97]	0.345
**Serum Folate (ng/mL)**	2.90 [2.10–3.38]	2.50 [1.82–3.48]	0.567
**CRP (mg/L)**	9 [5.08–16.20]	8.90 [3.60–12.95]	0.280
**IL-6 (pg/mL)**	4.50 [3.52–5.50]	4.80 [2.50–6.10]	0.994
**Liver function tests**			
**GGT (U/L)**	111 [50.15–179.12]	83.70 [40.05–128.88]	0.140
**ALT (U/L)**	42.25 [31.93–50.92]	44.45 [29.95–55.53]	0.556
**AST (U/L)**	34.45 [26.45–44.05]	32.95 [25.05–43.70]	0.891
**miRNA expression (relative units)**			
**miR-21 Expression**	1.54 [1.04–2.43]	1.52 [1.05–2.16]	0.840
**miR-34a Expression**	1.94 [1.28–2.19]	1.50 [1.10–2.08]	0.252
**miR-451a Expression**	2.06 [1.15–2.33]	1.50 [0.96–1.98]	0.144
**Categorical variables—n (%)**			
**Sex**			
**Female**	9 (50.0%)	13 (31.0%)	0.161
**Male**	9 (50.0%)	29 (69.0%)	0.161
**Ethnicity**			
**Caucasian**	17 (94.4%)	36 (85.7%)	0.334
**Other**	1 (5.6%)	6 (14.3%)	0.334
**Chronic Liver Disease**			
**No**	13 (72.2%)	35 (83.3%)	0.324
**Yes**	5 (27.8%)	7 (16.7%)	0.324
**Severity of Anemia**			
**Mild**	5 (27.8%)	16 (38.1%)	0.443
**Moderate**	6 (33.3%)	11 (26.2%)	0.574
**Severe**	7 (38.9%)	15 (35.7%)	0.815

Abbreviations: BMI, body mass index; MCV, mean corpuscular volume; RDW, red cell distribution width; WBC, white blood cell count; TIBC, total iron-binding capacity; CRP, C-reactive protein; IL-6, interleukin-6; GGT, gamma-glutamyl transferase; ALT, alanine aminotransferase; AST, aspartate aminotransferase; IQR, interquartile range. *p*-values generated by Mann-Whitney U test (continuous variables) and Chi-square or Fisher’s exact test (categorical variables), as appropriate.

**Table 3:** Predictors of Treatment Response—Binary Logistic Regression.

Variable	OR	95% CI Low	95% CI High	*p*-Value	Sig.
GGT	0.995	0.984	1.007	0.450	-
miR-451a Expression	0.777	0.278	2.174	0.631	-
Age	0.967	0.916	1.022	0.237	-
miR-34a Expression	0.764	0.259	2.253	0.625	-

AIC = 78.622 | BIC = 89.093 | Pseudo R^2^ (McFadden) = 0.064 | Hosmer-Lemeshow: χ^2^ = 4.162, df = 8, *p* = 0.842 (good fit). OR = Odds Ratio; CI = Confidence Interval. *p* < 0.05.

**Table 4:** Concise summary of the main findings.

Analysis	Increased/Higher	Decreased/Lower	No Significant Difference
Baseline severity groups	Years of alcohol consumption, average weekly alcohol intake, MCV, GGT, chronic liver disease frequency, miR-21, miR-34a, miR-451a	Hemoglobin, hematocrit	Age, BMI, Charlson Comorbidity Index, serum albumin, weight loss, RDW, reticulocyte count, WBC, platelets, iron, ferritin, TIBC, vitamin B12, folate, CRP, IL-6, ALT, AST, sex distribution
Treatment-response groups	No baseline variable was significantly higher in either the improved or no-change group	No baseline variable was significantly lower in either the improved or no-change group	Hemoglobin, hematocrit, MCV, RDW, reticulocyte count, WBC, platelets, iron indices, vitamin B12, folate, CRP, IL-6, GGT, ALT, AST, miR-21, miR-34a, miR-451a, anemia severity and chronic liver disease
miRNA correlation analysis	miR-21, miR-34a and miR-451a correlated positively with MCV, GGT and alcohol-exposure variables	miR-21, miR-34a and miR-451a correlated negatively with hemoglobin and hematocrit	No significant correlations were observed between serum vitamin B12 or folate and miR-21, miR-34a or miR-451a
Predictive analysis	No baseline predictor significantly predicted hematologic improvement	Not applicable	GGT, miR-451a, age and miR-34a were not significant predictors in logistic regression

Note: BMI, body mass index; MCV, mean corpuscular volume; RDW, red cell distribution width; WBC, white blood cell count; TIBC, total iron-binding capacity; CRP, C-reactive protein; IL-6, interleukin-6; GGT, gamma-glutamyl transferase; ALT, alanine aminotransferase; AST, aspartate aminotransferase. The table summarizes statistically significant and non-significant findings from the severity, treatment-response, correlation and predictive analyses.

## Data Availability

The raw data supporting the conclusions of this article will be made available by the authors on request.
